# Penetration of 0.3% ciprofloxacin, 0.3% ofloxacin, and 0.5% moxifloxacin into the cornea and aqueous humor of enucleated human eyes

**DOI:** 10.1590/1414-431X20175901

**Published:** 2017-07-03

**Authors:** G.C.M. Silva, V.A.P. Jabor, P.S. Bonato, E.Z. Martinez, S.J. Faria-e-Sousa

**Affiliations:** 1 Departamento de Oftalmologia, Faculdade de Medicina de Ribeirão Preto, Universidade de São Paulo, Ribeirão Preto, SP, Brasil; 2 Departamento de Física e Química, Faculdade de Ciências Farmacêuticas de Ribeirão Preto, Universidade de São Paulo, Ribeirão Preto, SP, Brasil; 3 Departamento de Medicina Social, Faculdade de Medicina de Ribeirão Preto, Universidade de São Paulo, Ribeirão Preto, SP, Brasil

**Keywords:** Ciprofloxacin, Eye banking, Eye drops, Fluoroquinolones, Moxifloxacin, Ofloxacin

## Abstract

We aimed to quantify the penetration of ciprofloxacin, ofloxacin, and moxifloxacin into the cornea and aqueous humor of cadaver eyes. A total of 60 enucleated eyes, not eligible for corneal transplantation, were divided into three groups and immersed in commercial solutions of 0.3% ciprofloxacin, 0.3% ofloxacin, or 0.5% moxifloxacin for 10 min. Whole corneas and samples of aqueous humor were then harvested and frozen, and drug concentrations analyzed by liquid chromatography tandem mass spectrometry. The mean corneal concentration of moxifloxacin was twice as high as ofloxacin, and the latter was twice as high as ciprofloxacin. The mean concentration of moxifloxacin in the aqueous humor was four times higher than the other antibiotics, and the mean concentrations of ciprofloxacin and ofloxacin were statistically similar. The amount of drug that penetrated the anterior chamber after a 10-min immersion was far below the safe limit of endothelial toxicity of each preparation. Moxifloxacin demonstrated far superior penetration into the cornea and anterior chamber of cadaver eyes compared to ciprofloxacin and ofloxacin. One should not expect endothelial toxicity with the commercial eye drops of ciprofloxacin, ofloxacin, and moxifloxacin that reach the anterior chamber through the cornea.

## Introduction

Eyes retrieved for corneal transplantation have a high rate of bacterial contamination that can lead to devastating graft infections, so it is essential for eye banks to minimize bacterial contamination (1,2). There are several protocols for reducing contamination of donor corneas, such as aseptic precautions during enucleation, disposal of eyes exposed to highly contaminated environments, use of corneal preservation solutions containing antibiotics, and soaking of whole eyeballs in antibiotic or antiseptic solutions (3,4).

Over the past 20 years, enucleated eyes harvested by the eye bank at Hospital das Clínicas de Ribeirão Preto have been immersed in 15 mL of 0.3% ciprofloxacin eye drops for 10 min before corneal removal. Despite this, the rate of bacterial colonization of corneoscleral buttons remains around 4%. In the literature, the percentage is even greater, leaving room for further improvement (4).

Other solutions of fluoroquinolones, such as 0.3% ofloxacin and 0.5% moxifloxacin, are also commercially available. Their efficacy and toxicity profiles resemble those of 0.3% ciprofloxacin but with greater ocular penetration (5,6). It is reasonable to assume that superior tissue penetration reduces bacterial contamination. However, data on eye penetration of these drugs have all been obtained under non-standardized conditions, most of them during surgeries of the anterior segment after the instillation of a few drops onto the corneal surface (7–15). This could partly explain the huge variability of reported results. We hypothesized, though, that our eye decontamination procedure could be an alternative to investigate the ocular penetration of ophthalmic topical drugs as, ultimately, trans-corneal penetration seems to occur primarily by passive diffusion (10).

In the absence of more specific information, we fully immersed cadaver eyes in commercial ophthalmic solutions of 0.3% ciprofloxacin, 0.3% ofloxacin and 0.5% moxifloxacin in order to quantify their penetration rates into the cornea and aqueous humor.

## Material and Methods

The experiment used 60 human eyeballs that did not meet the safety serological criterion for corneal transplantation. Only 1 eye of each donor was used. To be included in the study, the eyes could not have any of the following conditions: gross epithelial defects, gross alterations of transparency, congenital disorders, ectasias, corneal guttata, keratitis, and more than 72 h of preservation in the wet chamber. The study protocol was approved by the ethical board of our institution.

A set of numbers, ranging from 1 to 60, was randomly divided into three subsets of 20. Each eye received a number corresponding to its order of retrieval, which automatically allocated it to one of those subsets. The eyeballs were fully immersed for 10 min in 15 mL of one of the following commercial solutions: 0.5% moxifloxacin (Vigamox^©^, Alcon, Brazil), 0.3% ciprofloxacin (Ciloxan^©^, Alcon, Brazil), and 0.3% ofloxacin (Oflox^©^, Allergan, Brazil). The globes were then washed with 100 mL of saline to remove all traces of antibiotics. Next, whole corneas and samples of aqueous humor (collected with 30G tuberculin syringes at volumes of at least 100 µL) were collected and stored in non-transparent Eppendorf plastic vials. After identification, they were immediately stored in liquid nitrogen at –70°C until analysis by liquid chromatography tandem mass spectrometry (LC-MS/MS).

### Calibration solutions

Stock solutions of ciprofloxacin, ofloxacin, and moxifloxacin containing 200 μg/mL of the drugs were prepared in methanol acidified with 1% acetic acid. A 4.0 μg/mL cephalexin (internal standard; IS) solution was prepared in methanol. Calibration curves were obtained by analyzing spiked cornea samples in duplicate over the concentration range of 0.2–50 µg of the drug per gram of cornea or microliter of aqueous humor prepared by appropriate dilution of stock solutions. The results were plotted as a graph of peak area ratios versus analyte concentrations and the best relationship was obtained by linear least-squares regression analysis.

The thawed cornea samples were weighed and fully homogenized with 2 mL of water for 30 s (Omni TH tissue Homogenizer, USA), then spiked with 25 µL of IS, acidified with 200 µL of 5 mol/L hydrochloric acid, and extracted with 5 mL of dichloromethane:isopropanol mixture (9:1, v/v). The organic phases were collected and evaporated to dryness. The residues were dissolved in 40 µL of the mobile phase, and 20 µL of that was injected into the analytical column.

Aliquots of 1 µL of aqueous humor were spiked with 25 µL of IS and added to 0.9 mL of 0.1 mol/L phosphate buffer, pH 6.9, inside a polypropylene tube. The tubes were shaken for 15 min using a Vibrax VXR agitator (IKA, Germany) set at 1500 rpm and then centrifuged at 1800 *g* for 5 min at 4°C. Next, the organic product was extracted with 4 mL of dichloromethane by vigorously shaking in a vortex for 3 min and then centrifuging for 20 min (3500 *g* at 4°C). The organic layers (3 mL) were transferred to 10 mL conical glass tubes, and the solvent was evaporated to dryness under a stream of compressed air at room temperature. The resulting residues were dissolved in 100 µL of the mobile phase and vortex-mixed for 20 s. From that, 50 µL were analyzed by high performance liquid chromatography. Afterwards, the organic phase from each sample was transferred to conical glass tubes and the solvent was evaporated to dryness with a gentle stream of nitrogen. The ensuing residues were dissolved in 40 µL of the mobile phase, of which 20 µL were analyzed by liquid chromatography.

### Analysis of antibiotics in cornea and aqueous humor samples using LC-MS/MS

A Quattro LC triple quadrupole mass spectrometer (Micromass, UK) was interfaced via a Z-spray electrospray ionization (ESI) probe with a Shimadzu liquid chromatograph (Japan) equipped with an LC-AT VP solvent pump unit. A flow rate of approximately 0.2 mL/min was directed to the ESI probe by splitting the column effluent using a Valco tee connection. For LC-MS/MS analysis, the positive mode was chosen and the ion trap mode set at 2.5 kV. Nitrogen was used as a drying agent (380 L/h) and nebulizing gas (27 L/h), and argon was used as a collision gas. The cone voltage was 30 V for the three antibiotics and 10 V for cephalexin (IS). The collision energy was 25 eV for moxifloxacin and ciprofloxacin, 30 eV for ofloxacin, and 10 eV for the IS. Tandem mass spectrometry (MS/MS) detection was done by direct infusion of a standard solution (100 µg/mL) prepared in the mobile phase and introduced into the detector by an infusion pump at a flux rate of 20 µL/min. Samples containing ofloxacin, ciprofloxacin, moxifloxacin, and IS were separated on a reversed-phase LC XTerra¯ C18 column (100×3.9 mm, 3.5 µm particle size) using a C18 guard column (4×4 mm, 3.5 µm particle size). The mobile phase consisting of acetonitrile-water (30:70, v/v) plus 0.5% of glacial acetic acid was pumped at a flow rate of 0.5 mL/min under isocratic conditions. Quantification was performed by multiple reaction monitoring of the precursor ions and their corresponding product ions using a MassLynx data sampling and processing system (Micromass, version 4.1). The precursor-to-product ion transitions were monitored at m/z 332>231 for ciprofloxacin, m/z 402>384 for moxifloxacin, m/z 362>261 for ofloxacin, and m/z 348>157 for the IS.

### Statistical analysis

A linear random effects model was used to examine the relationship between the drug concentrations, antibiotics, and structures (cornea, µg/g; or aqueous humor, ng/µL) (16). We denoted *y_ijk_* as the concentration of the antibiotic *j* ( *j* = 1 for ciprofloxacin, *j* = 2 for ofloxacin, and *j* = 3 for moxifloxacin) observed in the structure *k* (*k* = 1 if cornea, and *k* = 2 if aqueous humor) of the eye *i* (*i* = 1,..., *n*). The statistical model was defined by log *y_ijk_* = *α*+*β_j_*+*γ_k_*+*δ_jk_*+*ω_i_*+*∊_ijk_*, where α is the intercept, *β_j_* is the effect of the antibiotics, *γ_k_* is the effect of the structures, *δ_jk_* are interaction effects between antibiotics and structures, and *ω_i_* are random effects (*i* = 1,..., 52 ), which were included in the model to account for the two measurements from each eye (each with a mean of zero and variance of $\sigma_{\omega }^{2}$). Finally, *∊_ijk_* are normal random errors with a mean of zero and variance of $\sigma_{j}^{2}$ where *j* = 1, 2, 3. This model is characterized as a heteroscedastic model, since the variances $\sigma_{j}^{2}$ are dependent on the antibiotics. The parameters of the model were estimated by a Bayesian approach using the Markov chain Monte Carlo methods implemented in OpenBUGS (GNU General Public License) software (17).

## Results

The advantages of the Bayesian statistical methods for analysis of ophthalmological data have been discussed in an article by Weiss (18). In Bayesian analysis of the proposed model, *β_j_*,*γ_k_* and *δ_jk_* were zero if *j* = 1 and/or *k* = 1, and non-informative prior distributions were used for all other parameters. The logarithms of the concentrations were chosen because of their skewed distributions. Consequently, the results are reported as geometric means with 95% credible intervals (CI), obtained from the parameter estimates (credible intervals are the Bayesian analogues of frequentist confidence intervals).

Table 1 presents the raw data. The arithmetic means in the bottom row of the table are only for comparison with similar data from the literature; they were not used for statistical analyses. Although twenty samples were collected from each eye, some were discarded for technical reasons, resulting in different sample sizes. Table 2 summarizes these data using geometric means, which tend to be smaller than arithmetic means. It answers the question: Once the mean drug concentration of a sample is known, what is the corresponding estimate for the entire population? The 95%CI is the range within which we expect to find the answer. For instance, given that the mean concentration of moxifloxacin in the corneal sample is 14.38 µg/g, the corresponding value for the whole population should be between 11.12 and 18.55 µg/g, with 95% certainty. The equivalent information for the aqueous humor is found in the bottom row of the table. The same rationale was used for the other antibiotics.

**Table 1.** Antibiotic concentrations in eyes immersed for ten minutes in three different solutions.

	0.3% Ciprofloxacin		0.3% Ofloxacin		0.5% Moxifloxacin	
	Cornea (µg/g)	Aq. humor (ng/µg)	Cornea (µg/g)	Aq. humor (ng/µg)	Cornea (µg/g)	Aq. humor (ng/µg)
	3.40	0.68	5.54	1.68	28.85	4.28
	3.22	0.91	10.37	1.42	36.84	4.36
	6.73	1.49	8.61	1.22	10.47	3.45
	2.92	0.82	13.00	-	15.70	2.66
	4.41	1.18	5.10	0.67	7.14	8.00
	3.37	0.96	3.02	1.35	19.25	4.75
	9.70	1.70	6.28	1.20	26.72	3.89
	2.95	1.84	4.71	1.11	14.88	4.29
	4.84	0.35	7.14	1.48	13.93	7.90
	2.64	0.37	3.52	-	10.71	4.48
	1.46	1.52	4.99	-	-	-
	-	0.81	-	-	15.17	5.93
	4.52	0.87	-	-	9.88	3.69
	5.22	0.44	5.06	1.46	9.88	6.55
	9.75	1.15	5.33	1.60	7.77	5.99
	1.87	1.83	8.79	1.77	-	-
	5.19	1.33	7.25	1.66	-	-
	-	1.32	8.53	1.92	-	-
	0.76	1.29	9.00	1.36	-	-
	1.73	1.86	8.91	1.36	-	-
Mean	4.15	1.14	6.95	1.42	16.23	5.02

Aq. humor: aqueous humor.

**Table 2.** Statistics summary of antibiotic concentrations in eyes immersed for ten minutes in three different solutions.

Structure/Antibiotic	n	Geometric mean	Std. Error	95%CI
Cornea (µg/g)				
0.3% Ciprofloxacin	18	3.46	0.512	(2.60–4.61)
0.3% Ofloxacin	18	6.48	0.613	(5.37–7.78)
0.5% Moxifloxacin	14	14.38	1.886	(11.12–18.55)
Aqueous humor (ng/µL)				
0.3% Ciprofloxacin	20	1.02	0.142	(0.78–1.34)
0.3% Ofloxacin	15	1.26	0.127	(1.02–1.53)
0.5% Moxifloxacin	14	4.78	0.621	(3.69–6.13)

Table 3 shows the ratios between the geometric mean concentrations of different pairs of antibiotics. It answers the question: In the population, how much more concentrated is one antibiotic over another? Again, the 95%CI is the range within which one expects to find the answer. For instance, given that the ratio between the geometric mean concentrations of moxifloxacin and ciprofloxacin in the corneal sample is 4.16, the related value for the whole population should be somewhere between 2.82 to 6.06, with 95% certainty. The same information for aqueous humor is found in the bottom row of the table. When a ratio between two numbers is one, they are equal. In the corneal samples, the 95%CIs of all ratios did not contain the number 1; so one could infer that there is a real difference among these ratios in the population. Likewise, since the two CIs of the moxifloxacin ratios in the aqueous humor samples do not contain the number 1, one can infer that the concentration of moxifloxacin in the population should be greater than that of ofloxacin and ciprofloxacin. Because the 95%CI of the ofloxacin/ciprofloxacin ratio in the aqueous humor contains the number 1, it is not possible to affirm that the aqueous humor concentrations of the two antibiotics differ in the population.

**Table 3.** Ratios between the geometric mean concentrations of pairs of antibiotics.

Structure/Antibiotic	Ratio between geometric means	Std. Error	95%CI
Cornea			
Ofloxacin/Ciprofloxacin	1.87	0.332	(1.32–2.63)*
Moxifloxacin/Ofloxacin	2.22	0.363	(1.62–3.06)*
Moxifloxacin/Ciprofloxacin	4.16	0.832	(2.82–6.06)*
Aqueous humor			
Ofloxacin/Ciprofloxacin	1.23	0.214	(0.88–1.71)
Moxifloxacin/Ofloxacin	3.81	0.632	(2.75–5.23)*
Moxifloxacin/Ciprofloxacin	4.68	0.907	(3.21–6.79)*

* P<0.05 (linear random effects model).

Table 4 summarizes the data concerning the ratios of the geometric mean concentrations of the antibiotics in the cornea and aqueous humor. This table was built to answer the following questions: In the population, does the antibiotic concentration in the cornea differ from that in the aqueous humor? If so, does drug partitioning vary with the antibiotic? Since none of the 95%CIs contained the number 1, the concentrations of the three fluoroquinolones were assumed to be greater in the cornea than in the aqueous humor. Because the differences among the mean ratios were not statistically significant, it was not possible to answer the second question.

**Table 4.** Ratios between the corneal and aqueous humor concentrations of antibiotics.

Ratio/Antibiotic	n	Geometric mean	Std. Error	95%CI
Cornea/Aqueous				
0.3% Ciprofloxacin	18	3.40	0.664	(2.33–4.94)
0.3% Ofloxacin	15	5.16	0.631	(4.04–6.54)
0.05% Moxifloxacin	14	3.01	0.502	(2.18–4.16)

## Discussion

We found striking differences in corneal concentrations of fluoroquinolones. The mean corneal concentration of moxifloxacin was twice as high as ofloxacin, which was twice as high as ciprofloxacin. Furthermore, moxifloxacin displayed a mean concentration four times higher than the other antibiotics in the aqueous humor. In contrast, the difference between ofloxacin and ciprofloxacin in the aqueous humor was not significant (Table 3). This suggested that ofloxacin had more difficulty reaching the anterior chamber than ciprofloxacin. However, we did not have enough data to validate this hypothesis, as the differences among the mean ratios in Table 4 were not statistically significant.

The immersion of enucleated eyes in antibiotic solutions opens up a new dimension for comparing the ophthalmic penetration rates of various medications. It is particularly suitable for contrasting the ratios of concentrations between different drugs under standardized conditions. Furthermore, drug partitioning between the cornea and aqueous humor can be tested under different circumstances, for instance by changing the time interval of immersion. This method seems to be more reliable than those that access drug penetration into the anterior chamber by instilling a few drops of the medication onto the cornea during surgeries of the anterior segment.

The limitation of the model is that the cadaver eye does not fully duplicate the living eye, with its tear film, lacrimal drainage, strong epithelial barrier, and slim stroma. However, we do not believe this should be a critical problem with eyes recently harvested and in good condition since the tissues are still alive. One should note that the eyes of our sample would have been eligible for corneal transplantation had they passed serological testing. Besides, trans-corneal penetration seems to occur primarily by passive diffusion (10). One can improve the protection of the epithelial integrity of the enucleated eyes by retrieving them in Hank’s buffered salt solution or phosphate-buffered saline (19).

By comparing the data of Table 2 to that of Mather et al. (20), who studied the susceptibility patterns of ciprofloxacin, ofloxacin, and moxifloxacin in the anterior segment of the eye, one can have an objective appraisal of our findings. In our study, the 95%CI for the mean concentration of ciprofloxacin ranged from 2.60 to 4.61 µg/g in the cornea, and 0.78 to 1.34 ng/µL in the aqueous humor. These values exceeded the minimum inhibitory concentrations (MICs) of most pathogens tested by Mather et al., both in the cornea and in the aqueous humor, except for fluoroquinolone-resistant (FQR) *Staphylococcus aureus* and FQR coagulase-negative staphylococci. The 95%CI for the mean concentration of ofloxacin ranged from 5.37 to 7.78 µg/g in the cornea, and 1.02 to 1.53 ng/µL in the aqueous humor. In the cornea, these values exceed the MICs for all the pathogens tested by Mather et al. (20), except for FQR staphylococci. However, in the aqueous humor, they were lower than the MICs of more than half of all the pathogens, including resistant staphylococci, *Streptococcus pneumoniae, Streptococcus viridans,* and *Enterococcus* sp. The 95%CI for the mean concentration of moxifloxacin ranged from 11.12 to 18.55 µg/g in the cornea, and 3.69 to 6.13 ng/µL in the aqueous humor. These values exceeded the MICs of all the pathogens reported by Mather et al. (20).

If these measurements would hold true *in vivo*, one could infer that the decreasing order of bactericidal efficacy for antibiotics in the cornea should be moxifloxacin, ofloxacin, and ciprofloxacin. In the aqueous humor, it should be moxifloxacin, ciprofloxacin, and ofloxacin. In either medium, moxifloxacin should be far more efficient.

Our results also suggest that one should not be concerned with endothelial toxicity when the fluoroquinolones reach the anterior chamber through the cornea. The amount of drug that penetrated the anterior chamber after a 10-min soaking was far below the safe limit of endothelial toxicity of each preparation (21–23). This information is particularly interesting to the eye banking practice.
